# Gene therapy with growth factors for periodontal tissue engineering–A review

**DOI:** 10.4317/medoral.17472

**Published:** 2011-12-06

**Authors:** Shaveta Sood, Shipra Gupta, Aneet Mahendra

**Affiliations:** 1 MDS Senior Assistant professor, Department of periodontics Dr H. S. Judge Institute of Dental Sciences and Hospital Panjab University Sector 25, Chandigarh, India; 2 MDS Associate Professor, Department of Periodontics, Dr H. S. Judge Institute Of Dental Sciences And Hospital Panjab University Sector 25, Chandigarh, India; 3 MD Associate Professor Department of Dermatovenereology, Mmimsr Mullana (Ambala), India

## Abstract

The treatment of oral and periodontal diseases and associated anomalies accounts for a significant proportion of the healthcare burden, with the manifestations of these conditions being functionally and psychologically debilitating. A challenge faced by periodontal therapy is the predictable regeneration of periodontal tissues lost as a consequence of disease. Growth factors are critical to the development, maturation, maintenance and repair of oral tissues as they establish an extra-cellular environment that is conducive to cell and tissue growth. Tissue engineering principles aim to exploit these properties in the development of biomimetic materials that can provide an appropriate microenvironment for tissue development. The aim of this paper is to review emerging periodontal therapies in the areas of materials science, growth factor biology and cell/gene therapy. Various such materials have been formulated into devices that can be used as vehicles for delivery of cells, growth factors and DNA. Different mechanisms of drug delivery are addressed in the context of novel approaches to reconstruct and engineer oral and tooth supporting structure.

** Key words:** Periodontal disease, gene therapy, regeneration, tissue repair, growth factors, tissue engineering.

## Concept

National Institute of Health defines tissue engineering as, an emerging multidisciplinary field involving biology, medicine, and engineering that is likely to revolutionize and improve the health and quality of life for millions of people worldwide by restoring, maintaining, or enhancing tissue and organ function. Periodontal tissue engineering deals with the repair of alveolar bone, tooth-associated cementum and periodontal ligament (PDL).

Dental tissue engineering is a relatively new field of reconstructive biology which utilizes mechanical, cellular or biologic mediators to facilitate regeneration of a particular tissue. It is now being successfully used for human application. This review article focuses on the emerging concepts and results of the application of biomaterials, growth factors and cell/gene therapy on the proteins, genes and cells to regenerate the lost periodontium. 

 Periodontal regeneration

**Figure 1:**
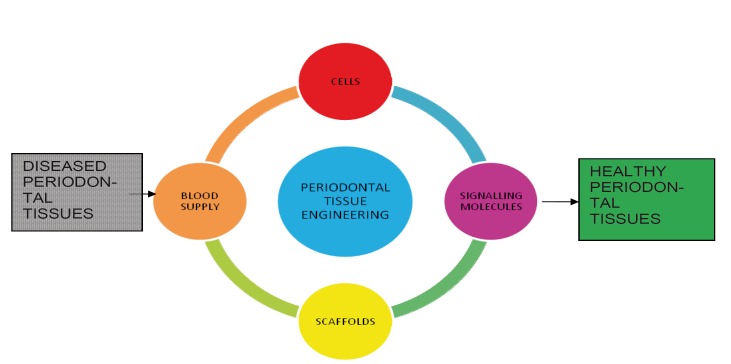
The Four Critical Elements Required In Periodontal Tissue Engineering.

Periodontal regeneration is defined as reproduction or reconstruction of a lost or injured part so that form and function of lost structures are restored. The regeneration of the periodontal tissues is de-pendent on four basic components. The appropriate signals, cells, blood supply and scaffold needed to target the tissue at the defect site (Fig 1) (1). All these elements play a fundamental role in the healing process and in the reconstruction of the lost tissue. The cells provide the machinery for new tissue growth and differentiation where as the growth factors or morphogens modulate the cellular activity and provide stimuli to the cells to differentiate and produce matrix for the developing tissue The new vascular networks provide the nutritional base for tissue growth and homeostasis. Finally, scaffolds guide and create a template structure three-dimensionally to facilitate the above processes required for tissue regeneration.

 Role of growth factors in periodontal tissue engineering

Growth factors are proteins that may act locally or systemically to affect the growth and function of cells in several ways. The application of growth factors to restore damaged tissues aims at regeneration through biomimetic processes, or mimicking the processes that occur during embryonic and post-natal development. The effects of growth factors in different phases of wound healing have been illustrated in ( Table 1) (2). 

 Platelet- derived growth factor (PDGF) was the first growth factor to be evaluated in preclinical periodontal and peri-implant regenerative studies. Proliferation, migration and matrix synthesis were observed on cultures of periodontal cells stimulated by PDGF, including gingival and PDL fibroblasts, cementoblasts, pre osteoblasts and osteoblastic cells. The PDGF family is com-posed of four growth factors: PDGF-A, -B, and the most recently discovered are PDGF-C and –D. All of these participate in the wound-healing process, but, until now, only the three isoforms PDGF-AA, BB and AB were evaluated in periodontal therapy. PDGF-BB is most effective on PDL cell mitogenesis and matrix biosynthesis (3).

According to Howell et al. (4), in a human Phase I/II clinical trial, PDGF/Insulin Growth Factor -I were considered safe when applied topically to periodontal osseous lesions, resulting in a significant improvement in bone growth and fill of periodontal defects, compared with standard therapy. According to Park et al. (5) and Cho et al. (6), PDGF alone was demonstrated in two preclinical studies in dogs in which alveolar bone defects of critical size were completely regenerated after treatment with PDGF-BB associated to guided tissue regeneration (GTR). The results were superior to the same treatment without PDGF. The authors concluded that PDGF stimulated formation of fibrous connective tissue in an early stage of repair, filling and stabilizing the wound. In a subsequent regenerative stage, the fibrous tissue was substituted with new bone and PDL.

**Table 1:**
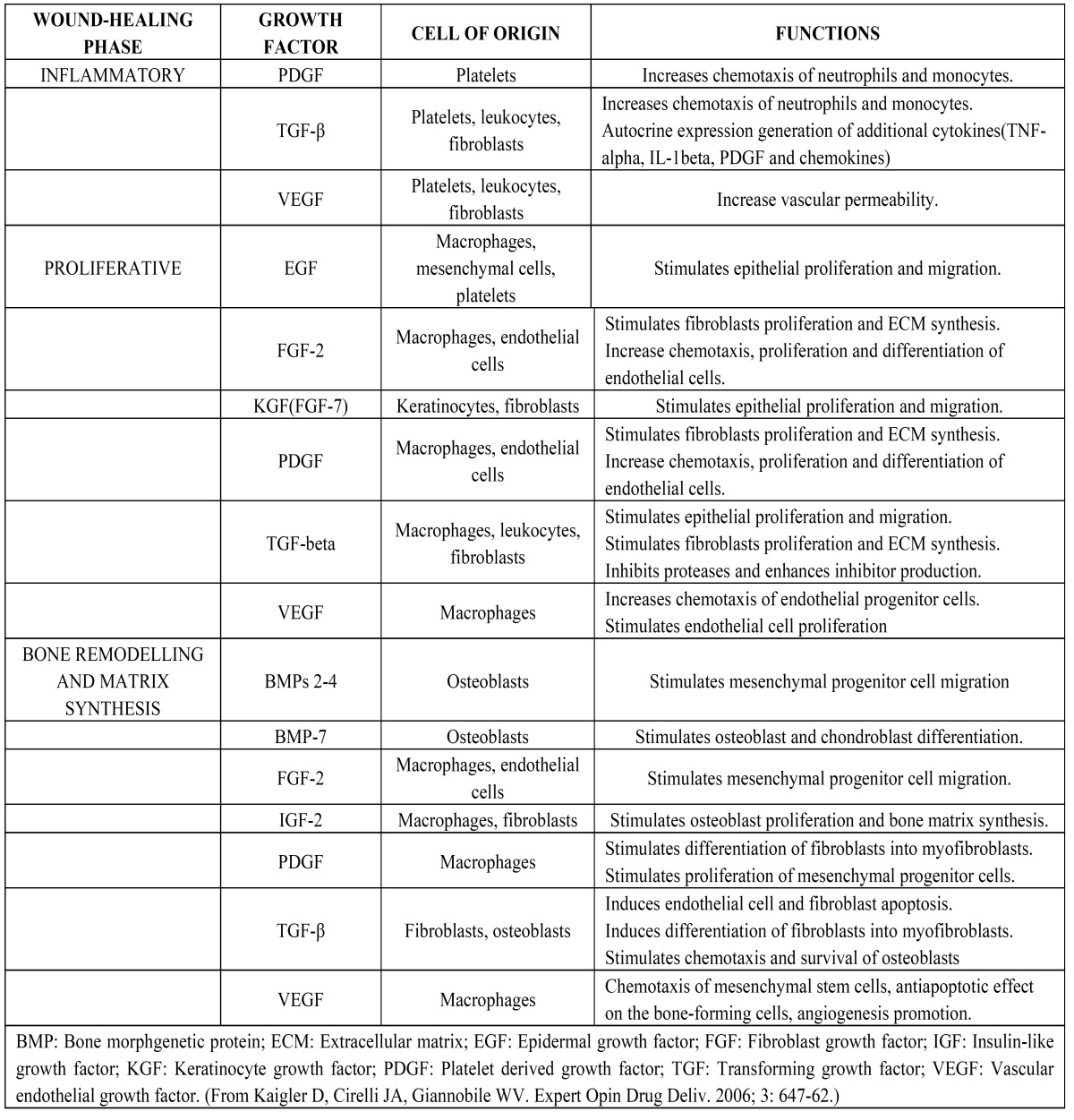
Effects of growth factors in the different phases of wound healing.

Wang et al. (7) showed enhanced fibroblast proliferation in early periodontal wound healing, after treatment of alveolar bone defects in dogs with PDGF. Camelo et al. (8) and Nevins et al. (9) conducted two studies using PDGF-BB with demineralised freeze-dried bone allograft in the treatment of different types of critical size periodontal bone defects. Histological sections provided evidence of periodontal regeneration.

Jayakumar et al. (10) conducted a multi-centre clinical trial on 54 patients with periodontal osse-ous defects. The patients were randomly assigned to rhPDGF-BB+β-TCP or β-TCP. Following periodontal surgery, respective implantation was performed. The primary and secondary end points of treatment were evaluated at the third and the sixth month. They reported a significant in-crease in the extent of linear bone growth and per cent bone fill over baseline in the rhPDGF-BB+β-TCP group when compared with the β-TCP group. Similarly, it also resulted in significantly higher area under the curve clinical attachment level gain at 0-6 months (p<0.01), CAL gain and greater reduction in probing depth at the third and the sixth month than that with β-TCP treat-ment alone. 

According to Takayama et al. (11) and Terranova et al. (12) the angiogenic and fibroblast stimulatory properties of Fibroblast growth factor (FGF-2) during wound healing and its chemotactic and proliferative effects on PDL cells, suggest its use for periodontal regenerative therapeutic ap-proaches. Many preclinical studies were conducted using this growth factor. Despite different concentrations of FGF-2 and different delivery systems used in these studies, all showed an improvement in the periodontal tissue regeneration, compared with control groups. These studies also suggested that its effects are dose dependent (13). 

Kitamura et al. (14) investigated the efficacy of the local application of recombinant human fibroblast growth factor-2 (FGF-2) in periodontal regeneration by conducting a double-blind, placebo-controlled clinical trial in 253 adult patients with periodontitis. Modified Widman periodontal sur-gery was performed, during which 200 μL of the investigational formulation containing 0% (vehicle alone), 0.2%, 0.3%, or 0.4% FGF-2 was administered to 2-or 3-walled vertical bone defects. Each dose of FGF-2 showed significant superiority over vehicle alone for the percentage of bone fill at 36 wks after administration, and the percentage peaked in the 0.3% FGF-2 group. They hence concluded that topical application of FGF-2 can be efficacious in the regeneration of human periodontal tissue that has been destroyed by periodontitis. 

 Transforming growth factor –β (TGF- β) is a multifunctional growth factor structurally related to bone morphogenetic protein but is functionally quite different. However, TGF-β can control gene expression either positively or negatively, a factor that can interfere with its therapeutic use (15). TGF-β1, the most abundant isoform of the TGF-β family and found primarily in the platelets and osseous tissue, has been used for this application. Clokie et al. suggested that TGF-β1 increased the amount of bone healing adjacent to dental implants in mini pigs (15). TGF-β1 seems to play an important role in inducing fibroblastic differentiation of PDL stem/progenitor cells and in maintaining the PDL apparatus under physiological conditions (16).

Markopoulou et al. (17) evaluated the in vitro effect of recombinant human transforming growth factor-beta 1 (rhTGF-β1) combined with two different bone grafts on human PDL (hPDL) cell differentiation. The hPDL cells were treated with TGF-β1 alone or in combination with a calcified freeze-dried bone allograft (FDBA) and a porous biphasic calcium phosphate (BC) bone graft. Cell differentiation effect was estimated by measuring alkaline phosphatase (ALPase) activity and osteocalcin secretion. Results demonstrated that rhTGF-β1 alone or in combination with FDBA and BC provoked a significant increase in ALPase activity as compared with controls. The findings of this study confirmed the beneficial role of rhTGF-β1 combined with FDBA and BC as carriers in periodontal regeneration.

Another important group of proteins for therapeutic applications are the Bone morphogenetic proteins (BMP). BMPs -2,-4, -7 and -12 have all been evaluated for periodontal and peri-implant bone regeneration. BMP-2 has been the most studied for bone and periodontal regenerative treatment (18). Several preclinical studies demonstrate significant improvement of alveolar bone re-gen-eration in different types of periodontal defects after treatment with rh BMP-2 via different carriers.

Another important therapeutic application of BMPs is for maxillary bone regeneration to allow replacement of lost teeth by os-seointegrated dental implants. This approach involves the re-generation of peri-implant bone after implant fixation or bone height improvement in areas below the maxillary sinus. Preclinical (19) and clinical (20) studies have shown improved bone formation after treatment with BMP-2. However, the use of different carriers and the association of barrier membrane (GTR technique) or other biomaterials seem to be critical factors in influencing the therapeutic outcome.

BMP-7 or osteogenic protein-1 is a potent modulator of osteogenesis and bone cell differentiation. Its effect in periodontal-regenerative treatment was evaluated in bony defects around tooth roots in preclinical studies. Significant improvement on bone and cementum regeneration was observed in dogs (21). An extensive cementogenesis was considered the most significant effect of BMP-7 in bony defects in baboons (22). Improvement of bone formation around titanium implants was also demonstrated by studies in animals (23).

The potential of BMP-12 to repair tendon and PDL tissues has been shown in vitro and in vivo stud-ies (24). A preliminary study in dogs compared rhBMP-12 with rhBMP-2 for the treatment of periodontal defects. The results showed less bone and more functionally oriented PDL between the new bone and new cementum after BMP-12 treatment, contrasting with a more parallel fiber arrangement of BMP-2-treated defects (25).

Moore YR, Dickinson DP, Wikesjo UM (26) conducted a comprehensive literature search to re-view the therapeutic effects of Growth/differentiation factor-5 (GDF-5), a member of the bone morphogenetic protein family. They concluded that GDF-5 appears to be a promising therapeutic agent for periodontal wound healing/regeneration as it supports/accelerates bone and ten-don/ligament formation in several musculoskeletal settings including periodontal tissues.

Kwon et al. (27) clinically evaluated the injectability, biocompatibility, safety, and periodontal wound healing/regeneration following application of a novel bioresorbable recombinant human growth/differentiation factor-5 (rhGDF-5)/ poly (lacticco-glycolic acid) (PLGA) construct. Bone formation showed apparent increased maturity (lamellar bone) at 6 weeks in sites receiving rhGDF-5/PLGA compared with the control. Both protocols exhibited significant increases in PDL, cementum, and bone regeneration over time. Increased bone formation was observed at sites receiving rhGDF-5/PLGA. They concluded that the rhGDF-5/PLGA construct appears to be a safe technology for injectable, ease-of-use application of rhGDF-5-stimulated periodontal wound heal-ing/regeneration. However, additional work to optimize the polymer carrier and rhGDF-5 release kinetics/dose might be required before evaluating the efficacy of this technology in clinical settings using minimally invasive approaches.

Though the results from preclinical and initial clinical studies using growth factors are encouraging, some limitations exist with respect to bone volume and predictability. Although in vitro studies have elucidated the role of growth factors in the cellular events of the different type of cells, several factors may influence the results in vivo. Limitations that restrict optimal responses of growth factor delivery include the short half-life of growth factors after being delivered in vivo. This may be due to proteolytic degradation, rapid diffusion and the solubility of delivery vehicles in chronic perio-dontal wounds. 

Amelogenins are a family of extracellular matrix proteins that regulate the initiation and growth of hydroxyappatite crystals during mineralization of the enamel. An acidic extract of enamel matrix derivative is being evaluated for clinical use. Enamel matrix derivative (EMD), uses proteins that are derived from embryonic enamel matrix with the purpose of mimicking the specific events that occur during the development of the periodontal tissues. EMD uses propylene glycol alginate as a vehicle in a viscous formulation. After coating the tooth root close to the periodontal defect, the propylene glycol alginate viscosity is reduced under physiological conditions and facilitates EMD release and precipitation. 

Trombelli et al. (28) reviewed clinical effects of the use of recombinant human platelet-derived growth factor-BB (rhPDGF-BB), platelet-rich plasma (PRP), commercially available enamel matrix derivative (cEMD) and peptide P-15 (P-15) (BAs) for the treatment of intra-osseous and furcation defects when used in addition to open flap debridement either alone or in association with guided tissue regeneration (GTR). They concluded that: (1) cEMD either alone or in combination with grafts can be effectively used to treat intra-osseous defects and the clinical results appear to be stable long term; (2) the additional use of a graft seems to enhance the clinical outcome of cEMD; (3) the combined use of rhPDGF-BB and P-15 with a graft biomaterial has beneficial effects in intra-osseous defects.

Esposito et al. (29) conducted a systematic review to test whether EMD is effective, and to com-pare EMD versus GTR, and various bone grafting procedures for the treatment of intrabony defects. They reported that after one year of application of EMD, there was significant improvement in the probing attachment levels (1.1 mm) and reduced pocket depths (0.9 mm) when compared to a placebo or control, however, the high degree of heterogeneity observed among trials suggested that the results have to be interpreted with great caution. In addition, a sensitivity analysis indicated that the overall treatment effect might be overestimated. The actual clinical advantages of using EMD are unknown. With the exception of significantly more postoperative complications in the GTR group, there was no evidence of clinically important differences between GTR and EMD. Bone substitutes may be associated with less gingival recession than EMD. 

 Growth factor delivery for periodontal tissue engineering

The concepts of GTR and guided bone regeneration have been used to retard apical migration of epithelial cells favoring the healing by cells from the PDL region and adjacent alveolar bone. Thus, different membranes are used in these procedures as barriers. The development of both naturally de-rived and synthetic materials allows the degradation properties to be controlled by the composition and ratios of compounds that comprise the material. The advantage is that, with the more recently developed polymers, these properties can be tailored to meet the specific needs of the application as to how long one desires to have the material maintained.

The tissue-engineering strategies involve the use of different polymer systems to serve as synthetic extracellular matrices. These polymers serve as delivery depots not only for growth factors and DNA molecules, but also for cells. Cells can be delivered after ex vivo expansion and combined with biomaterials to continually proliferate and differentiate into new tissues. Successful regeneration and engineering of a wide variety of oral structures have been demonstrated with this cell transplantation approach, and these tissues include bone, PDL, oral mucosa, skin and teeth. Cells can also be genetically modified ex vivo prior to implantation, creating a combined gene therapy–cell transplantation approach. 

Two common types of polymeric materials used in growth factor delivery strategies are natural collagen-derived materials and synthetic polymers of lactic and glycolic acid (i.e., poly (lactidecoglycolide)). Extracellular matrix-derived macromolecules such as collagen have been used for many years in biomaterial application, and it is now possible to create artificial analogues of ex-tracellular matrix proteins using recombinant DNA technology. Collagen is degraded by cells within regenerating tissue, and biodegradable synthetic polymers such as poly (lactidecoglycolide) are hydrolysed into natural metabolites, lactic acid and glycolic acid by the action of water at regenerated sites. The poly (lactidecoglycolide) scaffolds are popularly employed due to familiarity with their functional properties and uses in other applications (e.g., biodegradable sutures). Additionally, growth factors and DNA can be incorporated into these materials and released in a controlled, sustained manner to enhance tissue regeneration. 

A variety of new injectable materials such as hydrogels are also being developed for growth factor delivery applications. These injectables are especially attractive because, in clinical application, they can allow for minimally invasive delivery of inductive molecules. As an example, alginate hydrogels bearing cell-adhesion ligands have been used as scaffolds for cell encapsulation and transplantation, and have yielded promising results in experiments directed toward the engineering of bone tissue (7). Another group of polymers are the biomimetic polymers. These polymers combine the information content and multifunctional character of natural materials with the mechanical properties of synthetic polymers. This hybrid concept has been used in the binding of polymers with specific amino acids (such as the tripeptide sequence RGD) that are capable of regulating cell adhesion. 

Another area of increasing attention has been the development of shape-memory materials that have one shape at one temperature and another shape at a different temperature. These materials have the ability to memorize a permanent shape that can be substantially different from an initial temporary shape. As an example, a bulky device could potentially be introduced into a surgical site as a temporary shape (such as a string or freely flowing material), penetrate through a small area of the site, and then be expanded in response to different cues into a permanent shape (i.e., a stent or a sheet). The response signals that stimulate the changes in shape in response to environmental cues are incorporated within the material during its fabrication. These materials have been designated as ‘smart’ materials, having the ability to appropriately change their structural and functional material properties in response to environmental cues. These materials have also demonstrated great promise and the ability to control their built-in signaling is what makes them attractive for growth factor delivery strategies (30). 

 Gene therapy for periodontal tissue regeneration

Gene therapy may achieve greater bioavailability of growth factors within periodontal wounds, which may provide greater regenerative potential. Gene therapy involves the transfer of genetic information to target cells, which enables them to synthesize a protein of interest to treat a disease. The preferred strategy for gene transfer depends on the required duration of protein release and the morphology of the target site.

Gene transfer is accomplished through the use of viral and nonviral vectors. Examples of viral vectors are retroviruses, adenoviruses (Ad) and adeno-associated viruses (AAV), and nonviral vectors include plasmids and DNA polymer complexes. Retroviruses introduce RNA together with two enzymes, called reverse transcriptase and integrase, into the target cell. Initially, the reverse transcriptase enables the production of a DNA copy from the retrovirus RNA molecule. Subsequently, the integrase adds the DNA copy into the target cell DNA. When the genetically altered host cell divides later, its descendants contain the modified DNA. Because the integrase enzyme may insert the DNA copy into an arbitrary position of the target cell DNA, gene disruption and uncontrolled cell division (that is, cancer) may occur (1). 

Ad contains DNA, which is introduced into the target cell and subsequently transferred into its nucleus. In contrast to the fate of the retrovirus DNA copy, the Ad-DNA is not incorporated into the host cell’s genetic material. Consequently, when the Ad-infected target cell divides later, its descendants are not genetically altered, nor do they contain the Ad-DNA genetic material. AAV derived from parvovirus family, are small viruses with a single-stranded DNA genome that causes no known human dis-eases. The AAV infects dividing and non dividing cells by integrating its genetic material on chromosomes of the target cell. Types of recombinant AAV have been developed either to remain extra chromosomal or integrate into nonspecific chromosomal sites. Research has demonstrated that the AAV can be used to correct genetic defects in animals. One disadvantage of the AAV is that it is small and possesses the capacity to carry no more than usually two genes. 

Nonviral alternatives do not have the drawbacks of undesired host immune reactions or potential tumorigenesis so they likely would be given more consideration in the future. Plasmids and DNA polymer complexes carry the genetic information in the form of DNA to express a foreign protein. Various design features of nonviral delivery of DNA include chromosomal integration and their ability to alter gene expression. 

Various gene delivery methods are available to administer growth factors to periodontal defects for tissue engineering (2). The delivery method can be tailored to the specific characteristics of the wound site. For example, a horizontal one- or two-walled defect may require the use of a supportive carrier, such as a scaffold where as other defect sites may be conducive to the use of an Ad vector embedded in a collagen matrix. The most important point is the risk associated with the use of gene therapy in periodontal tissue engineering. Studies that examine the use of specific delivery methods and DNA vectors in periodontal tissue engineering reflect the aim to maximize the duration of growth factor expression, optimize delivery method to periodontal defect, and minimize patient risk.

## Complementary Explorations

 Preclinical studies that evaluate growth factor gene therapy for tissue engineering

To overcome the short half-lives of growth factor peptides in vivo, gene therapy that uses a vector that encodes the growth factor is utilized to stimulate tissue regeneration. The two main strategies of gene vector delivery have been applied to periodontal tissue engineering. Gene vectors can be introduced directly to the target site (in vivo technique) (31) or selected cells can be harvested, expanded, genetically transduced, and then reimplanted (ex vivo technique). In vivo gene transfer involves the insertion of the gene of interest directly into the body anticipating the genetic modification of the target cell. Ex vivo gene transfer includes the incorporation of genetic material into cells exposed from a tissue biopsy with subsequent reimplantation into the recipient. 

 Platelet-derived growth factor gene delivery

The PDGF-gene transfer strategies have been used in tissue engineering to improve healing in soft tissue wounds, such as skin lesions. 

Chen et al. (32) were able to demonstrate the prolonged effects of Ad delivery of PDGF for the better understanding of sustained PDGF signaling. Ad encoding PDGF-β transduced gingival fibroblasts and enhanced defect fill by induction of human gingival fibroblast migration and proliferation. On the other hand, continuous exposure of cementoblasts to PDGF-α had an inhibitory effect on cementum mineralization, possibly via the upregulation of osteopontin and subsequent enhancement of multinucleated giant cells in cementum-engineered scaffolds. Ad/PDGF-1308 (a dominant-negative mutant of PDGF) inhibited mineralization of tissue-engineered cementum possibly because of downregulation of bone sialoprotein and osteocalcin with a persistence of stimulation of multinucleated giant cells. These findings suggested that continuous exogenous delivery of PDGF-α may delay mineral formation induced by cementoblasts, whereas PDGF clearly is required for mineral neogenesis .

Jin et al. (31) demonstrated that direct in vivo gene transfer of PDGF-B stimulated tissue regeneration in large periodontal defects. Descriptive histology and histomorphometry revealed that human PDGF-B gene delivery promotes the regeneration of cementum and alveolar bone, whereas PDGF-1308, a dominant-negative mutant of PDGF-A, has minimal effects on periodontal tissue regeneration.

 Bone Morphogenetic protein gene delivery

Lieberman et al. (33) demonstrated gene therapy for bone regeneration, they transduced the bone marrow stromal cells with rhBMP-2 to form bone within an experimental defect comparable to skeletal bone. Baltzer et al. (34) showed regeneration of skeletal bone by directly administering Ad5/BMP-2 into a bony segmental defect in rabbits. Further advances in the area of orthopedic gene therapy using viral delivery of BMP-2 have provided further evidence for the ability of in vivo and ex vivo bone engineering. Franceschi et al. (35) investigated in vitro and in vivo Ad gene transfer of BMP-7 for bone formation. Ad transduced nonosteogenic cells also were found to differentiate into bone-forming cells and produce BMP-7 or BMP-2 in vitro and in vivo. Study by Huang et al. (36) using plasmid DNA encoding for BMP-4 with a scaffold delivery system was found to enhance bone formation when compared with blank scaffolds. 

In an early approach to regenerate alveolar bone in an animal model, the ex vivo delivery of Ad-encoding murine BMP-7 was found to promote periodontal tissue regeneration in large mandibular periodontal bone defects (37). BMP-7 gene transfers not only enhanced alveolar bone repair but also stimulated cementogenesis and PDL fiber formation. Of interest, the alveolar bone formation was found to occur via a cartilage intermediate. When genes that encoded the BMP antagonist nog-gin were delivered, inhibition of periodontal tissue formation resulted. A recent study by Dunn et al. (38) showed that direct in vivo gene delivery of Ad/BMP-7 in a collagen gel carrier promoted successful regeneration of alveolar bone defects around dental implants. These experiments provide promising evidence that shows the feasibility of in vivo and ex vivo gene therapy for periodontal tissue regeneration and peri-implant osseointegration.

 Angiogenic factors for periodontal repair

The blood supply has a key role on the nutrition of newly engineered tissues. However, a major chal-lenge in periodontal regeneration is the targeting of angiogenesis to an avascular tooth root surface. Basic fibroblast growth factor (bFGF or FGF-2) has been demonstrated to have potent angiogenic activity and potential to induce the growth of immature PDL cells. The mRNA level of laminin in PDL cells, which plays an important role in angiogenesis, is up regulated by FGF-2 stimulation. Thus it may in turn accelerate periodontal regeneration.

Enamel Matrix Derived protein (EMD) has angiogenic effects both in vitro and in vivo. The more rapid initial healing may not be directly influenced by the angiogenic effect of EMD alone. At least two other mechanisms probably contribute to the acceleration of wound healing. First, PDL cells se-crete growth factors, including TGF-β1, IL-6, and PDGF-AB after exposure to EMD. TGF-β1 and PDGF-AB have been shown to accelerate the rate of healing in periodontal wounds by specifically stimulating the proliferation of PDL cells. Second, it has been demonstrated that EMD can modulate the bacterial growth of putative periodontal pathogens (Actinobacillus actinomycetemcomitans, Porphyromonas gingivalis, and Prevotella intermedia), but not the normal flora during periodontal wound healing. 

The technical challenges confronting the tissue engineering of vasculature are many. First is the selection of appropriate vascular cells and scaffold materials. The majority of studies to date typically involve in vitro culturing of bone marrow cells or smooth muscle cells in combination with a collagen-based matrix until a tubular structure is formed, thus allowing endothelial cells to attach to the vessel wall. Scaffolds need to be designed to support the proper formation of vascular tissue and possess the mechanical properties that can match those of native arteries. The synthetic vessel must withstand the fluid shear stress and strain from blood flow and have adequate burst strength to withstand physiological blood pressures. Finally; incompatibilities between synthetic engineered grafts and native blood vessels must be quantified and evaluated.

Well-defined discriminating preclinical models followed by well-designed clinical trials are needed to further investigate the true potential of these growth and differentiation factors (39). All current or emerging paradigms have either been shown to have limited and variable outcomes or have yet to be developed for clinical use. To accelerate clinical translation, there is an ongoing need to develop therapeutics based on endogenous regenerative technology (ERT), which can stimulate latent self-repair mechanisms in patients and harness the host’s innate capacity for regeneration. ERT in periodontics applies the patient’s own regenerative ‘tools’, i.e. patient-derived GFs and fibrin scaffolds, sometimes in association with commercialized products (e.g. Emdogain and Bio-Oss), to create a material niche in an injured site where the progenitor/stem cells from neighboring tissues can be re-cruited for in situ periodontal regeneration (40). 

## Conclusion

A review of literature on the use of growth regulatory molecules along with gene therapy permits a model to consider approaches to oral tissue engineering. Developments in polymeric and ceramic scaffolding systems for cell, protein and gene delivery have undergone significant growth. The targeting of signaling molecules or growth factors (via proteins or genes) to the periodontium has lead to significant new knowledge regarding use of bioactive molecules to promote cell proliferation, differentiation, matrix biosynthesis, and angiogenesis. For improvements in the outcomes in periodontal regenerative medicine, scientists will need to examine dual delivery of host modifiers or anti-infective agents to optimize the results of therapy. For future growth and development in the field of oral tissue –engineering, combination of several multi disciplinary approaches including engineer-ing, dentistry and medicine will be needed.
